# Supplementary material for: A modular microfluidic platform to study how fluid shear stress alters estrogen receptor phenotype in ER+ breast cancer cells
Source: Microsyst Nanoeng. 2024 Feb 16;10:25. doi: 10.1038/s41378-024-00653-0 (PMC10873338; doi:10.1038/s41378-024-00653-0)
Supplement: Supplementary file 1 — Supporting Information [file 41378_2024_653_MOESM1_ESM.docx]

**Supporting Information for**

**A modular microfluidic platform to study how fluid shear stress alters estrogen receptor phenotype in ER^+^ breast cancer cells.**

**Braulio Andrés** **Ortega Quesada^1,2^, Jonathan Cuccia^3^, Rachael Coates^3^, Blake Nassar^1^, Ethan Littlefield^3^, Elizabeth C. Martin^4*^, Adam T. Melvin^1,2*^.**

*^1^Cain Department of Chemical Engineering, Louisiana State University, Baton Rouge, LA, 70803*

*^2^Department of Chemical and Biological Engineering, Clemson University, Clemson, SC, 29634*

*^3^Biological and Agricultural Engineering, Louisiana State University, Baton Rouge, LA, 70803*

*^4^Department Medicine, Section Hematology and Medical Oncology, Tulane University, New Orleans, LA, 70118*

**Corresponding authors*

**Table of Contents**

[Supplemental methods 3](#_Toc150452084)

[Computational modeling of fluid flow in the modular microfluidic device 3](#_Toc150452085)

[Supplemental Figures 4](#_Toc150452086)

[Figure S1. Fluid shear stress profile in the microwell trapping array. 4](#_Toc150452087)

[Figure S2. Tracking of single cells to measure their position at different frames 5](#_Toc150452088)

[Figure S3. Exposure to FSS does not result in differences in ERK1/2 phosphorylation between sheared and non-sheared populations. 6](#_Toc150452089)

[Figure S4. Single cell count of stained cells during on-chip experiment investigating the role of FSS exposure on Akt phosphorylation 7](#_Toc150452090)

[Figure S5. Distribution of the on-chip single data obtained during p-AKT immunostaining 8](#_Toc150452091)

[Figure S6. On-chip analysis of MCF-7 cells exposed to FSS exhibit mTOR phosphorylation 9](#_Toc150452092)

[Figure S7. On-chip analysis of MCF-7 cells exposed to FSS exhibit Signal Transducer and Activator of Transcription-3 phosphorylation 10](#_Toc150452093)

[Figure S8. Single cell count of stained cells during on-chip experiment investigating the role of FSS exposure on p-ERα Ser167 11](#_Toc150452094)

[Figure S9. Proposed mechanism for activation of cell proliferation and survival through AKT and mTOR phosphorylation 12](#_Toc150452095)

[Supplemental Tables 13](#_Toc150452096)

[Table S1. Experimental measurements of the position of single cells at different time points to determine their velocity when they are being sheared in the 70x100 um shearing device with a flowrate of 7 uL/min. 13](#_Toc150452097)

[Table S2. The average experimental velocity of single cells when they are being sheared at 10 dyn/cm2 in the 70x100 um shearing device 14](#_Toc150452098)

[Supplemental Videos 14](#_Toc150452099)

[Video S1. Cells being sheared at 10 dyn/cm2 in the 70x100 um shearing device 14](#_Toc150452100)

# Supplemental methods

## Computational modeling of fluid flow in the modular microfluidic device

COMSOL Multiphysics 5.3 with the computational fluid dynamics (CFD) module was used to model the velocity profile and the fluid shear stress profile into the shearing device and microwell trapping array. Water fluidic settings were used since the viscosity and density of the media used during shearing experiments are very similar to water. The following parameters were specified: 70 μm for the width, 100 μm for the height, 1 m long channel, 7 μL/min of flowrate, and non-slip boundary conditions for the first shearing device used at 10 dyn/cm^2^. For the second shearing device used to shear at 20 dyn/cm^2^ the parameters were 100 μm for the width, 150 μm for the height, 1.5 m long channel, 48 μL/min of flowrate, and non-slip boundary conditions. The velocity profile was obtained for the different geometries and FSS magnitudes were calculated for a cell at different positions across the cross-sectional area of the channel (e.g. at the center and on the edges).

**Variant of pressure drop equation of Hagen-Poiseuille in rectangular channels.**

$$\Delta P_{channel}=\frac{\alpha\mu QL}{WH^{3}}$$

Where

- $\Delta P_{channel}$= pressure drop in the channel
- $Q$ = flowrate
- $L$ = channel length
- $W$ = channel width
- $\mu$ = fluid viscosity
- $\alpha= 12\left[ 1-\frac{192H}{\pi^{5}W}\tanh\left( \frac{\pi W}{2H} \right) \right]^{-1}$

# Supplemental Figures


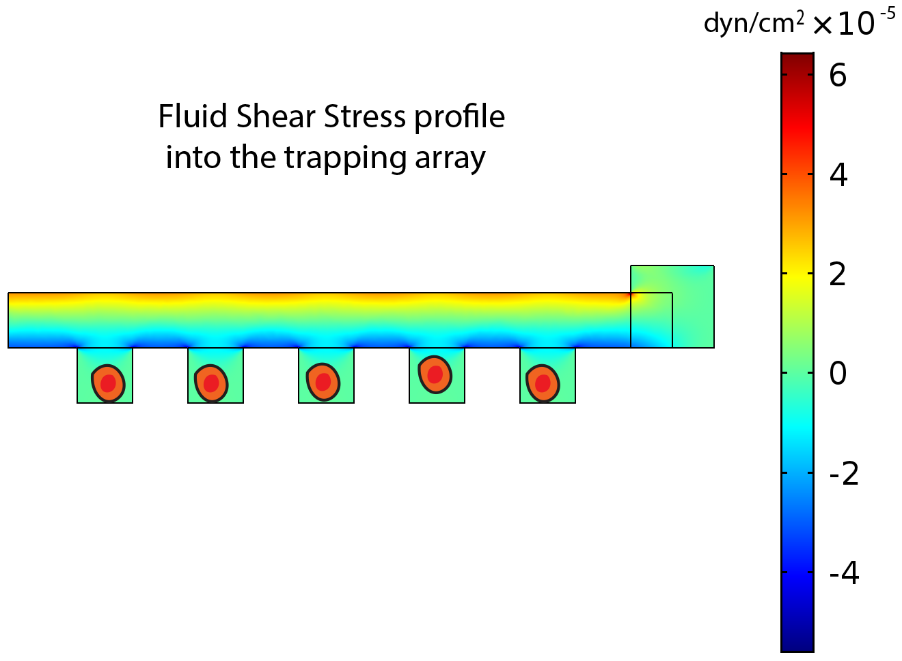


Figure S1. Fluid shear stress profile in the microwell trapping array. Side view simulation modeling the fluid flow profile in the microwell trapping array channel and traps. Simulations confirm that trapped cells are exposed to negligible FSS magnitudes during washing and immunostaining steps.


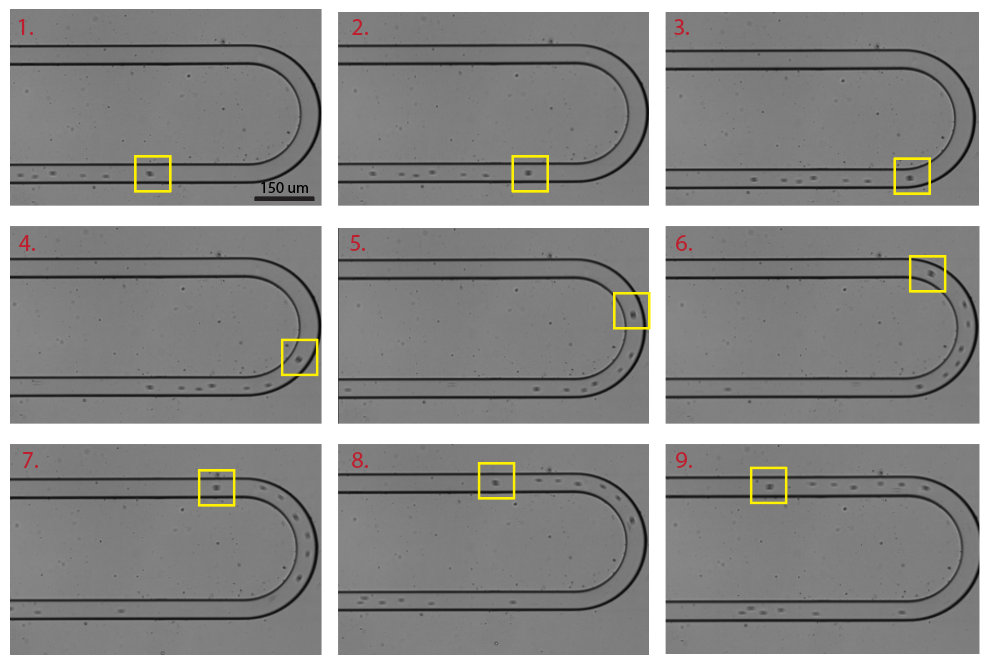


Figure S2. Tracking of single cells to measure their position at different frames**.** Frames taken every 12.37 ms show how single cells flow at an average velocity of 0.018 m/s in the 70x100 um serpentine when the flowrate is 7 uL/min


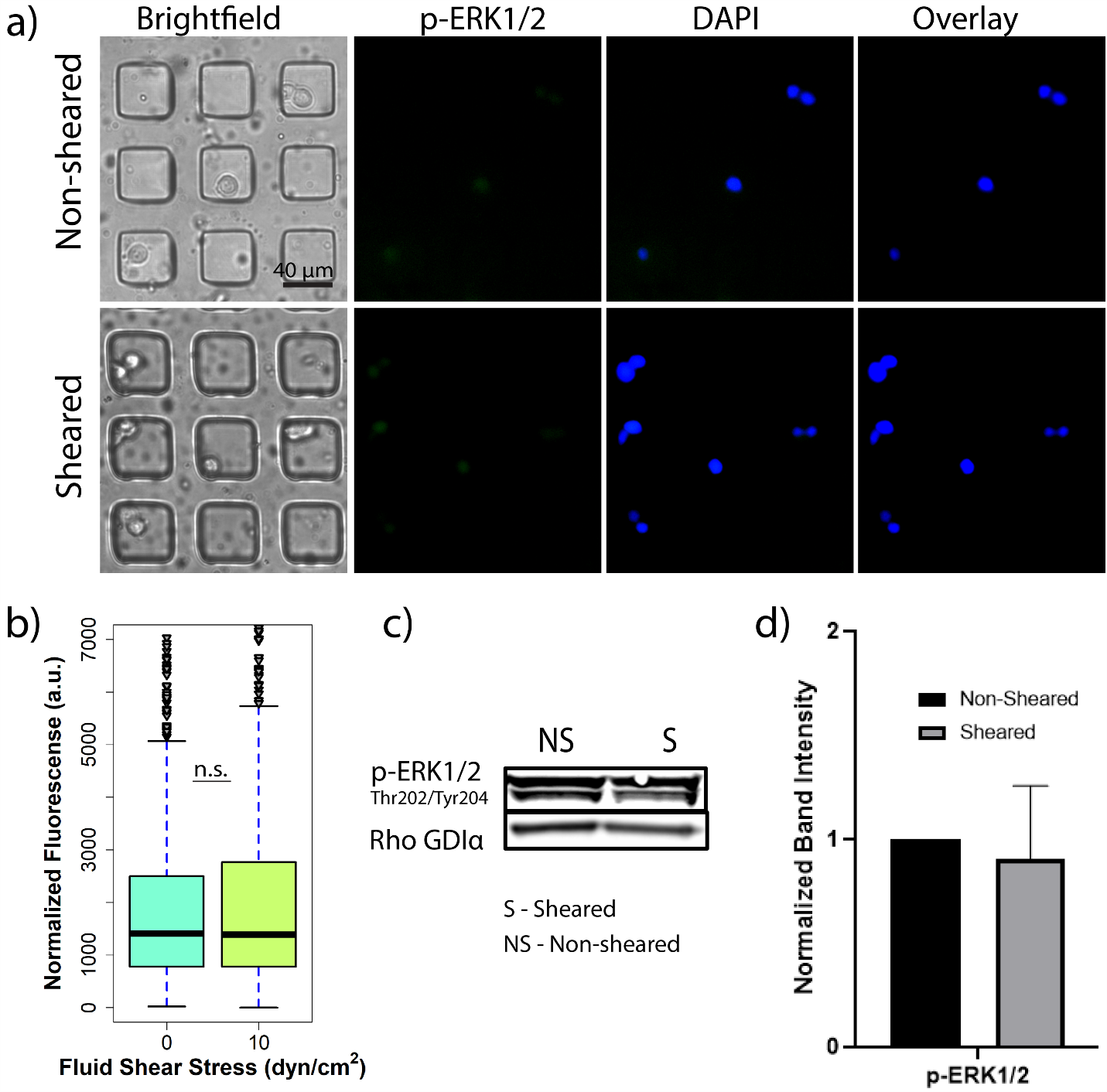


Figure S3. Exposure to FSS does not result in differences in ERK1/2 phosphorylation between sheared and non-sheared populations. A) Representative immunostaining images of single MCF-7 cells exposed to shear at a magnitude of 10 dyn/cm^2^ for ERK1/2 Thr202/Tyr204 phosphorylation (green) and DAPI (nuclear, blue). B) Quantification and distribution of normalized fluorescence in single cells coupled with one-way ANOVA showing no statistically significant changes between the sheared and non-sheared populations of MCF-7 cells. C) Western blot essay for p-ERK1/2(Thr202/Tyr204). D) Graphical representation of the normalized band density. The amount of phosphoprotein was normalized to Rho GDIα and the amount of phosphoprotein from the non-Sheared population was standardized at 1. Off–chip data is representative of n=2 biological replicate. (*** indicates statistically significant p<0.001, * indicates statistically significant p<0.05, ns indicates statistically non-significant p>0.05).


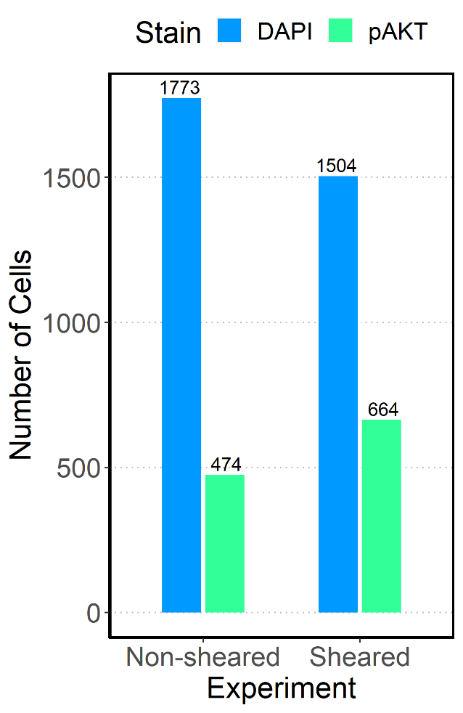


Figure S4. Single cell count of stained cells during on-chip experiment investigating the role of FSS exposure on Akt phosphorylation**.** Single stained cells were counted to compare the number of cells with phosphorylated AKT (at Ser473) against the total number of stained cells in the device using a DAPI counterstain.


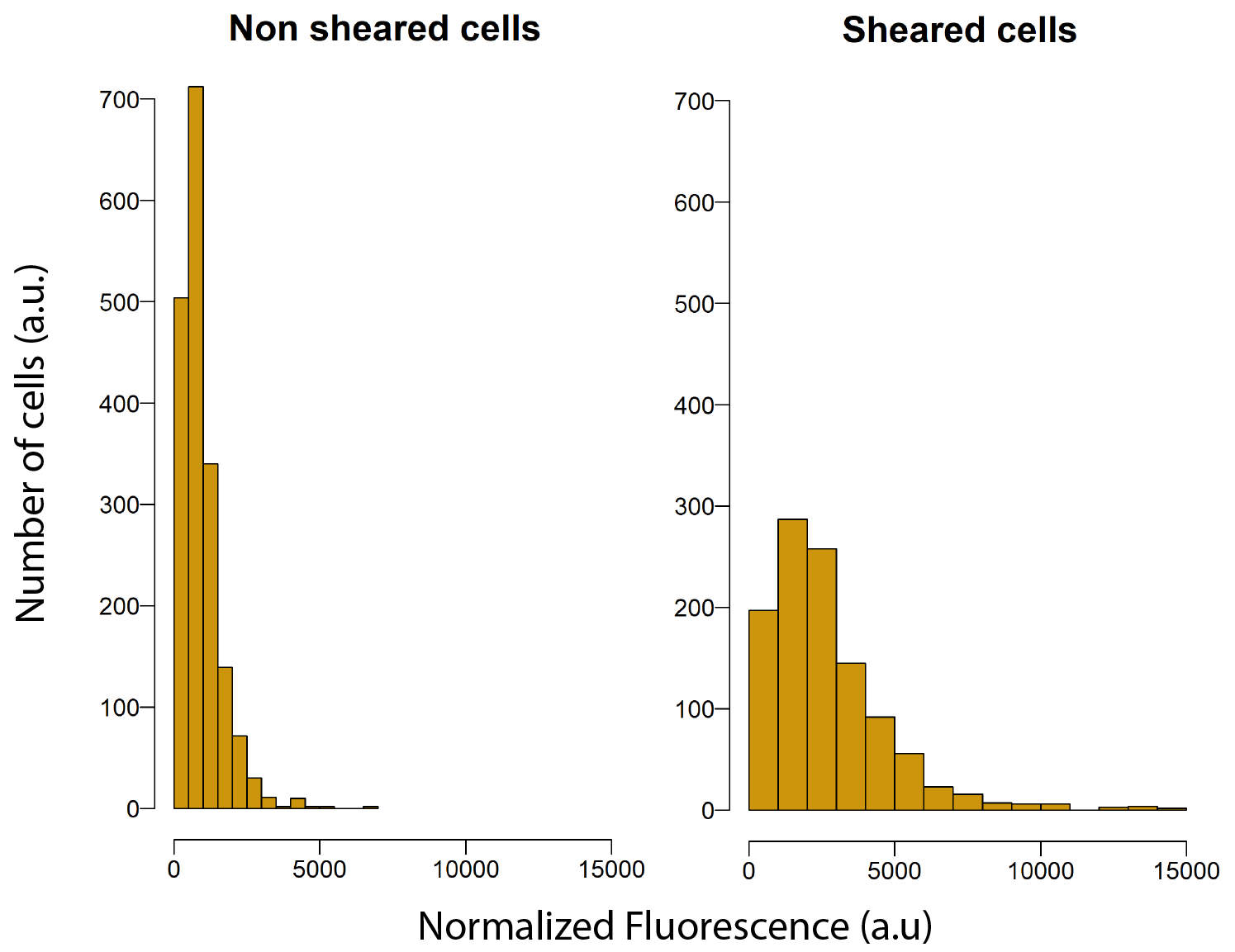


Figure S5. Distribution of the on-chip single data obtained during p-AKT immunostaining**.** The fluorescent signal of sheared and non-sheared populations for p-AKT Ser473 that was measured through ImageJ to obtain the distributions was grouped and then distributed in 14 groups in each population to plot the frequency chart. Data shows the non-sheared population has the data moved to the left, which means majority of the cells with low fluorescent intensities.


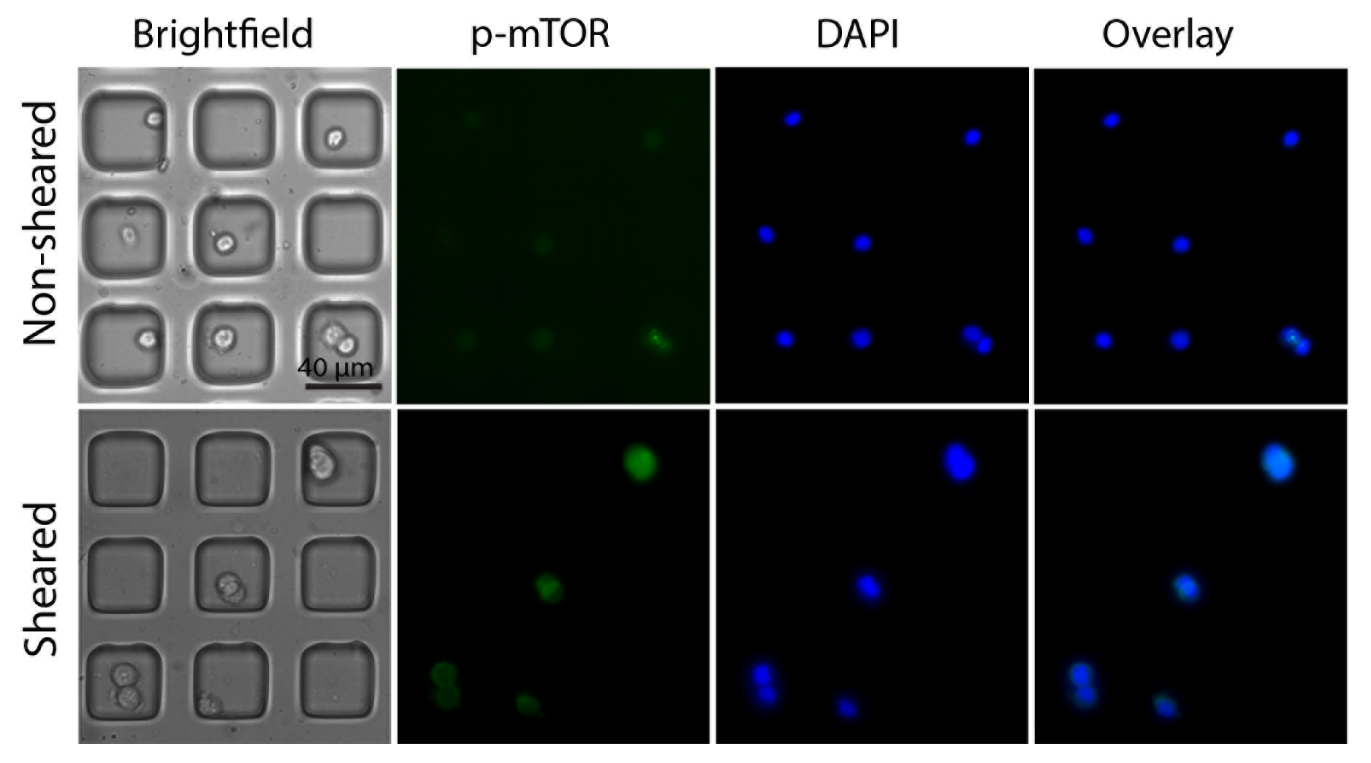


Figure S6. On-chip analysis of MCF-7 cells exposed to FSS exhibit mTOR phosphorylation**.** Representative immunostaining images of single MCF-7 cells exposed to shear at a magnitude of 10 dyn/cm^2^ for mTOR Ser2448 phosphorylation (survival, green) and DAPI (nuclear, blue).


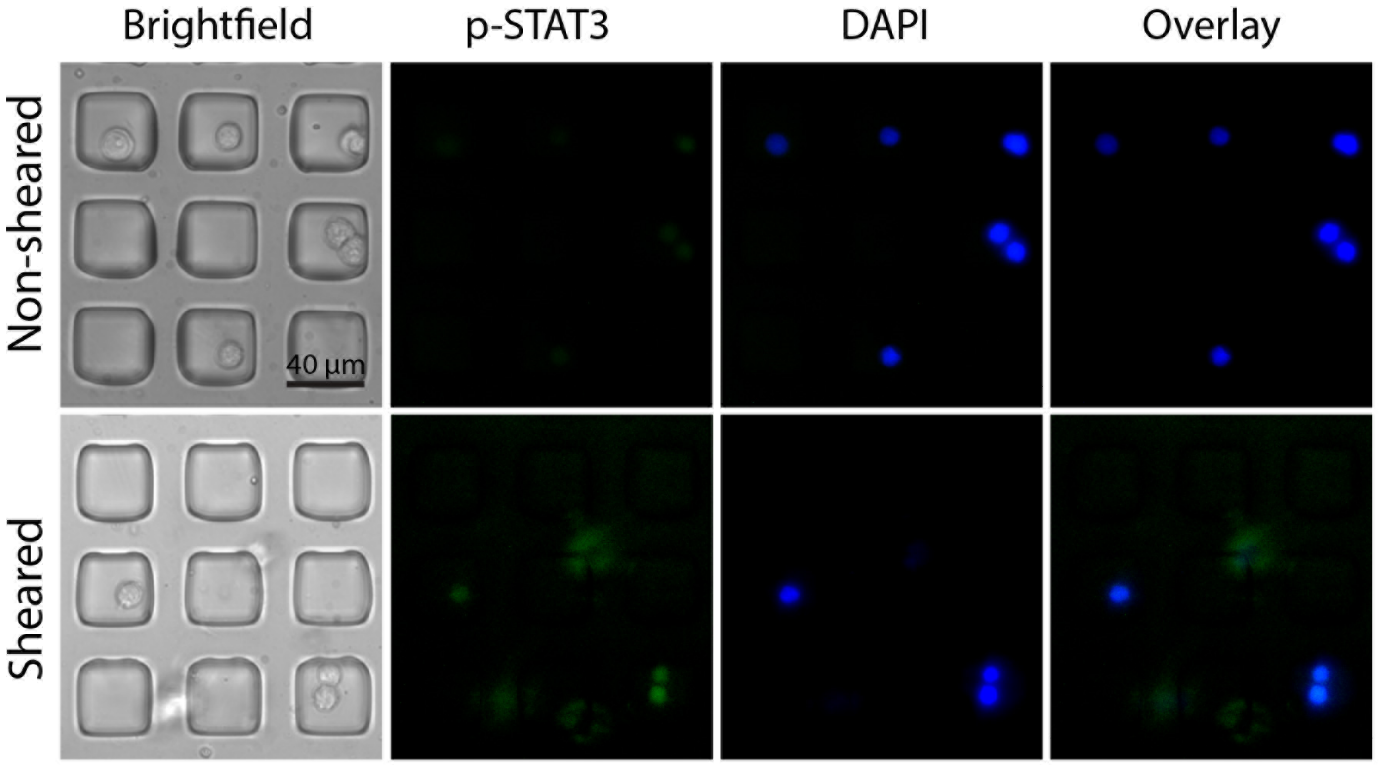


Figure S7. On-chip analysis of MCF-7 cells exposed to FSS exhibit Signal Transducer and Activator of Transcription-3 phosphorylation**.** Representative immunostaining images of single MCF-7 cells exposed to shear at a magnitude of 10 dyn/cm^2^ for STAT3 Tyr 705 phosphorylation (green) and DAPI (nuclear, blue).


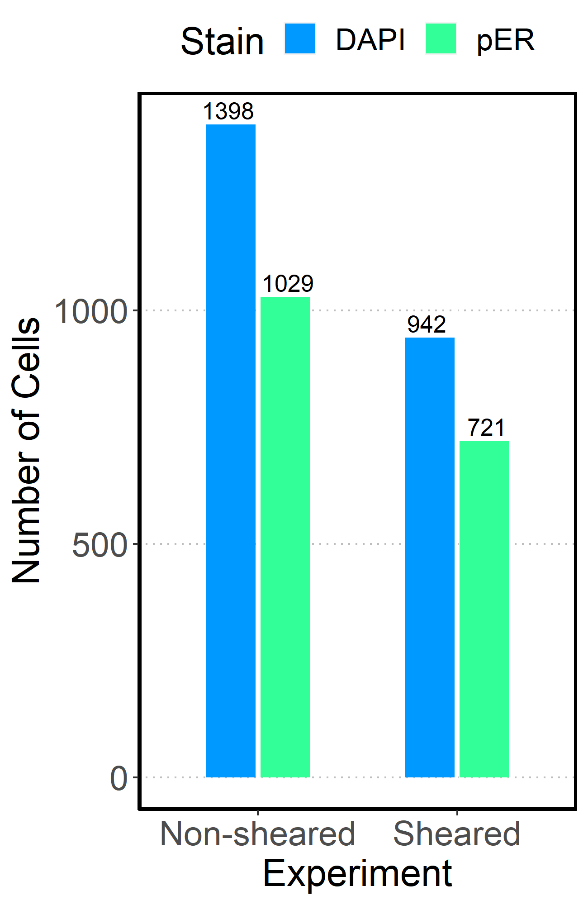


Figure S8. Single cell count of stained cells during on-chip experiment investigating the role of FSS exposure on p-ERα Ser167**.** Single stained cells were counted to compare the number of cells with phosphorylated ERα (atSer167) against the total number of stained cells in the device using a DAPI counterstain.


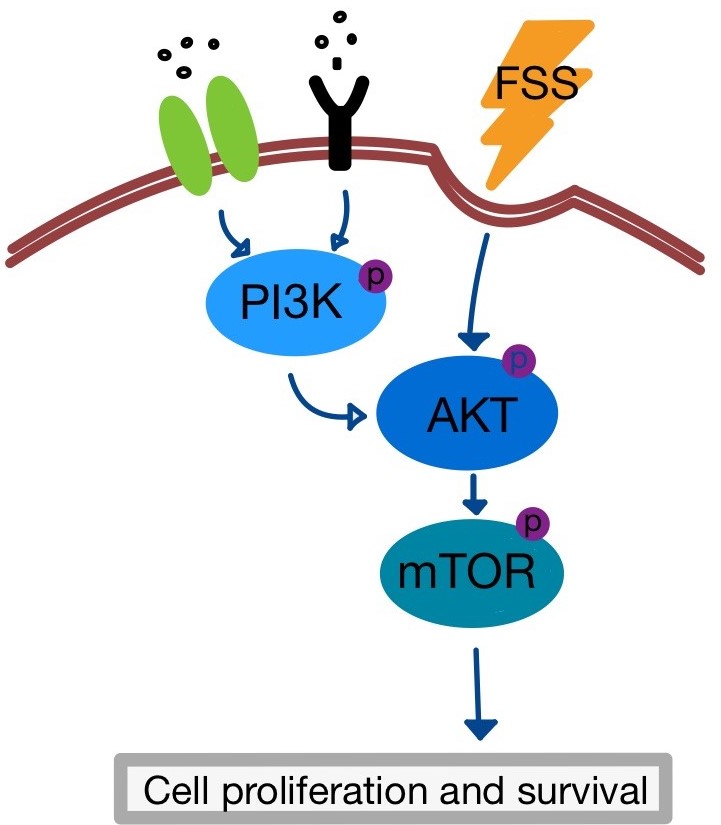


Figure S9. Proposed mechanism for activation of cell proliferation and survival through AKT and mTOR phosphorylation**.** Acquisition of the proliferative phenotype in ER+ breast cancer cells through AKT and mTOR phosphorylation due to exposure to FSS

# Supplemental Tables

## Table S1. Experimental measurements of the position of single cells at different time points to determine their velocity when they are being sheared in the 70x100 um shearing device with a flowrate of 7 uL/min.

|  | **Time (ms)** | **Distance (um)** | **Velocity (m/s)** |
| --- | --- | --- | --- |
| **Cell 1** | 0.00 | 231.40 | 0.0187 |
|  | 12.38 | 223.60 | 0.0181 |
|  | 24.75 | 224.90 | 0.0182 |
|  | 37.13 | 214.54 | 0.0173 |
|  | 49.51 | 214.52 | 0.0173 |
|  | 61.88 | 221.00 | 0.0179 |
|  | 74.26 | 214.52 | 0.0173 |
|  | 86.64 | 219.70 | 0.0178 |
|  | 111.39 | 210.64 | 0.0170 |
|  | 123.77 | 224.90 | 0.0182 |
| **Cell 2** | 0.00 | 222.30 | 0.0180 |
|  | 12.38 | 211.90 | 0.0171 |
|  | 24.75 | 224.90 | 0.0182 |
|  | 37.13 | 221.90 | 0.0179 |
|  | 49.51 | 218.40 | 0.0176 |
|  | 61.88 | 213.20 | 0.0172 |
|  | 74.26 | 232.70 | 0.0188 |
|  | 86.64 | 232.72 | 0.0188 |
|  | 111.39 | 226.20 | 0.0183 |
|  | 123.77 | 222.40 | 0.0180 |
| **Cell 3** | 0.00 | 234.00 | 0.0189 |
|  | 12.38 | 232.72 | 0.0188 |
|  | 24.75 | 235.30 | 0.0190 |
|  | 37.13 | 231.40 | 0.0187 |
|  | 49.51 | 222.32 | 0.0180 |
|  | 61.88 | 228.80 | 0.0185 |
|  | 74.26 | 239.20 | 0.0193 |
|  | 86.64 | 234.00 | 0.0189 |
|  | 111.39 | 221.00 | 0.0179 |

Table S2. The average experimental velocity of single cells when they are being sheared at 10 dyn/cm2 in the 70x100 um shearing device.

| **Cell** | **Average (m/s)** |  | **Standard Deviation (m/s)** |
| --- | --- | --- | --- |
| 1 | 0.017773 | ± | 0.000518 |
| 2 | 0.017991 | ± | 0.000567 |
| 3 | 0.018662 | ± | 0.000484 |

# Supplemental Videos

Video S1. Cells being sheared at 10 dyn/cm2 in the 70x100 um shearing device.
